# Altered BOLD Response during Inhibitory and Error Processing in Adolescents with Anorexia Nervosa

**DOI:** 10.1371/journal.pone.0092017

**Published:** 2014-03-20

**Authors:** Christina Wierenga, Amanda Bischoff-Grethe, A. James Melrose, Emily Grenesko-Stevens, Zoë Irvine, Angela Wagner, Alan Simmons, Scott Matthews, Wai-Ying Wendy Yau, Christine Fennema-Notestine, Walter H. Kaye

**Affiliations:** 1 Department of Psychiatry, University of California San Diego, La Jolla, California, United States of America; 2 Veterans Affairs San Diego Healthcare System, San Diego, California, United States of America; 3 Department of Radiology, University of California San Diego, La Jolla, California, United States of America; Bellvitge Biomedical Research Institute-IDIBELL, Spain

## Abstract

**Background:**

Individuals with anorexia nervosa (AN) are often cognitively rigid and behaviorally over-controlled. We previously showed that adult females recovered from AN relative to healthy comparison females had less prefrontal activation during an inhibition task, which suggested a functional brain correlate of altered inhibitory processing in individuals recovered from AN. However, the degree to which these functional brain alterations are related to disease state and whether error processing is altered in AN individuals is unknown.

**Methodology/Principal Findings:**

In the current study, ill adolescent AN females (n = 11) and matched healthy comparison adolescents (CA) with no history of an eating disorder (n = 12) performed a validated stop signal task (SST) during functional magnetic resonance imaging (fMRI) to explore differences in error and inhibitory processing. The groups did not differ on sociodemographic variables or on SST performance. During inhibitory processing, a significant group x difficulty (hard, easy) interaction was detected in the right dorsal anterior cingulate cortex (ACC), right middle frontal gyrus (MFG), and left posterior cingulate cortex (PCC), which was characterized by less activation in AN compared to CA participants during hard trials. During error processing, a significant group x accuracy (successful inhibit, failed inhibit) interaction in bilateral MFG and right PCC was observed, which was characterized by less activation in AN compared to CA participants during error (i.e., failed inhibit) trials.

**Conclusion/Significance:**

Consistent with our prior findings in recovered AN, ill AN adolescents, relative to CA, showed less inhibition-related activation within the dorsal ACC, MFG and PCC as inhibitory demand increased. In addition, ill AN adolescents, relative to CA, also showed reduced activation to errors in the bilateral MFG and left PCC. These findings suggest that altered prefrontal and cingulate activation during inhibitory and error processing may represent a behavioral characteristic in AN that is independent of the state of recovery.

## Introduction

Anorexia nervosa (AN) is characterized by severe emaciation, a relentless drive for thinness, and distorted body image. AN typically has a narrow range of age of onset (early adolescence), a relatively stereotypic presentation of symptoms, and tends to be female gender specific. It often has a chronic and relapsing life-threatening course [1]–[3], with the highest death rate of any psychiatric illness [4]. There is no proven treatment that reverses symptoms [5] or FDA approved medication [6]–[8]; improving our understanding and treatment of AN is therefore of immense clinical and public health importance. Clinically, pure restrictor-type AN individuals are often over-controlled, over-concerned about consequences, and perfectionistic [9]–[12]. They also tend to be anhedonic and ascetic, able to sustain self-denial of food as well as most comforts and pleasures in life [13]. Although the understanding of the pathophysiology of AN and other eating disorders has lagged behind other major psychiatric disorders, a growing body of evidence suggests that AN is a neurobiologically based disorder characterized by alterations in neurocircuitry supporting inhibition and cognitive control [9], [14]–[21].

Inhibitory control and error monitoring are critical executive functions involved in regulating behavior and emotions. Both cognitive inhibition (i.e., the suppression of previously activated cognitive processes) and behavioral inhibition (i.e., delaying gratification, inhibiting motor responses or resisting impulses) require intact cognitive control [22]. An impaired ability to overcome inhibition or switch behaviors may underlie symptoms in people with AN [23], [24]. Cognitive and neuropsychological tests reveal that AN individuals have an enhanced ability to delay monetary reward [25] and are impaired in cognitive set-shifting [26]–[34] as evidenced by elevated perseverative errors, although findings for impaired set-shifting in adolescent AN are mixed [35]–[42]. This enhanced cognitive control and ability to delay reward may help to maintain persistent food restriction and is thought to result from altered functioning of neurocircuitry governing inhibitory control.

Neuroimaging studies in healthy participants show that widely distributed and partially overlapping brain systems regulate inhibitory and error processing. Response inhibition involves a dorsal executive system that includes the dorsal anterior cingulate cortex (ACC), the dorsolateral prefrontal cortex (DLPFC) – comprised of the middle frontal gyrus (MFG), inferior frontal cortex, and premotor cortex – the inferior parietal lobule, and the caudate nucleus [43]–[46]. In particular, the dorsal ACC, which has extensive reciprocal connections with the DLPFC [47] and the dorsal caudate [48], monitors behavior in potential conflicts [49]–[52]. This neural circuit has been implicated in tasks requiring conflict resolution and the suppression of a learned response in favor of an alternate response (e.g., WCST, Flanker task, Simon Spatial Incompatibility, Go/No-Go, and stop signal tasks). The error processing system, which is responsible for monitoring performance, involves the rostral ACC and adjoining medial prefrontal cortex, the left and right insular cortex and the left precuneus/posterior cingulate [53]. While subregions of the ACC are differentially associated with inhibitory and error processing, transitional regions between them [54] permit the integration of these processes for cognitive control.

Recent studies in adult AN reveal increased activity within dorsal executive circuitry associated with impaired set-shifting, and reduced prefrontal activation during error monitoring and motor inhibitory control. Behavioral evidence also suggests that dorsolaterally- and medial-frontally-mediated executive functions may be differentially affected in AN [55]. For example, ill AN adults performing a set-shifting paradigm during fMRI showed greater activation of dorsolateral frontoparietal networks during task shift trials, which is thought to be indicative of excessive effortful and supervisory cognitive control [19]. Conversely, ill AN adults showed reduced dorsal ACC response to commission errors on a flanker task [56], and blunted cingulate function in relation to executive function [57]. Ill AN adults also showed reduced error monitoring demonstrated by reduced EEG error-related negativity in the context of improved performance [56], which suggests that hypoactivity of the ACC does not necessarily lead to diminished task performance. A possible explanation for this intact performance may be related to recruitment of other brain areas in order to increase cognitive control. Similarly, a combined group of ill AN restricting and binge/purge subtypes showed decreased ventrolateral prefrontal cortex activation during set shifting error feedback trials of the Wisconsin Card Sorting Test, indicating altered response to errors when shifting cognitive set [18]. During a motor response Go/No-Go task, ill restricting-type AN adolescents showed reduced DLPFC activation compared to a binge eating/purging group on No-Go (i.e., successfully inhibited) vs. go trials [20]; whereas during an affective Go/No-Go task using food and non-food stimuli, AN showed reduced putamen activity compared to healthy peers [16]. Together, these results suggest that individuals with AN require fewer inhibitory resources to maintain behavioral performance, as more experience with a task can lead to reduced activation [58]. Notably, when required to inhibit responses to affectively rewarding stimuli (e.g., pictures of physical activity), an elevated medial prefrontal inhibitory BOLD response was seen in AN participants [16], consistent with Zastrow et al’s [19] finding of an elevated brain response to task switching and calling into question the impact of increasing inhibitory demand on neural correlates of inhibitory control in AN.

Prior work by our group has demonstrated that adult individuals recovered from AN relative to comparison participants with no history of AN showed less prefrontal activation during stop signal task (SST) trials that required participants to inhibit a motor response (e.g., button press; similar to a No-Go trial on a Go/No-Go task) [17]. In that study, we examined the effect of increased inhibitory demand on brain activity in recovered AN participants and healthy comparison participants by parametrically manipulating the timing between when an auditory (Stop) signal was presented relative to a Go signal. Shorter delays between the Go and Stop signals resulted in less difficult trials, (i.e., easier to successfully stop the button press), whereas longer delays resulted in more difficult trials, (i.e., harder to successfully stop the already initiated button press). Using a voxel-wise analysis, we observed that adult women who were recovered from AN relative to controls showed less activation within the prefrontal cortex, including the MFG, during hard inhibit trials but similar prefrontal activity during easy inhibit trials (when inhibitory demand was low). These findings suggested a demand-specific modulation of inhibitory control circuitry in recovered AN adults, whereby recovered AN adults may require the engagement of fewer inhibitory resources (i.e., less PFC activation) to maintain inhibitory performance as inhibitory load is increased. However, it has yet to be established whether these findings extend to adolescents ill with AN, an age closer to the onset of the disorder.

In the present study, adolescent females currently ill with AN restricting-type and healthy comparison participants performed the SST during fMRI. We hypothesized that, similar to adults recovered from AN, currently ill AN adolescents will show less activation during inhibitory processing. Thus, our first goal was to extend our previous findings in adults recovered from AN [17] to adolescents currently ill with AN. Replicating this finding in ill AN adolescents would support the notion that altered inhibitory processing represents a behavioral characteristic of AN rather than a marker of the state of illness. Second, we hypothesized that ill AN adolescents would exhibit altered functional brain activity during error processing. This would extend our prior findings in recovered adults by adding the examination of inhibition accuracy at both the behavioral and neural response level. To accomplish these goals, we used a region of interest analysis approach to examine brain response in a priori hypothesized regions known to be involved in inhibitory control and error processing: specifically, the middle frontal gyrus (e.g., DLPFC), the anterior cingulate, and the posterior cingulate. Finally, we explored associations between brain response during inhibition and perseverative error during the Wisconsin Card Sorting Test [59], a behavioral measure of cognitive flexibility and part of our neuropsychological testing battery. We expected that perseverative errors would be negatively correlated with the BOLD response to error processing, reflecting an impaired ability to process errors. Overall, evidence for altered functional brain responses during inhibitory and error processing would support our overarching hypothesis that the ability to inhibit consummatory drives may be associated with neural processes underlying elevated self-control in AN (e.g., altered dorsal cognitive circuit function).

## Methods

### Participants

Twelve adolescent females aged 12–18 and meeting DSM-IV criteria for restricting-type AN within six months of study participation were recruited from the UCSD Eating Disorder (ED) Treatment and Research Program, and were receiving Family-Based Therapy [60] at study entry. Participants reported consuming ∼75–100% of their prescribed daily caloric needs at the time of the study. Twelve age-matched healthy comparison adolescent (CA) females were recruited through local advertisements. Axis I diagnoses were made by a child and adolescent psychiatrist with expertise in adolescent ED; assessments used included the Mini International Neuropsychiatric Interview for Children and Adolescents (MINI-KID) [61], and a modified Module H (ED diagnosis) from the Structural Clinical Interview for DSM-IV Axis I Disorders [62] that included additional questions to further define ED characteristics. Exclusion criteria for all participants included: past history of alcohol or drug abuse or dependence within three months of study enrollment; serious medical or neurological concerns; and any condition contraindicative to magnetic resonance imaging. Two participants with AN were on olanzapine but one of these participants was subsequently excluded from group analyses due to motion artifact during the fMRI scan. The CA and their first-degree relatives had no history of an ED. The study was conducted according to the protocol approved by the Institutional Review Board of the University of California, San Diego. Participants under the age of 18 gave written informed assent, and their parents gave written informed consent; participants aged 18 gave written informed consent. Participants completed other diagnostic and clinical assessments at a separate session occurring, on average, 24.8 days (S.D. = 26.3) prior to the imaging session (Beck Depression Inventory [BDI], Temperament Character Inventory [TCI], State-Trait Anxiety Inventory [STAI] described elsewhere [63], as well as the Similarities and Matrix Reasoning subtests of the Wechsler Abbreviated Scale of Intelligence [64], the Reading subtest of the Wide Range Achievement Test Revision 4 [65], and the Wisconsin Card Sorting Test (WCST) [59]). Between group comparisons of assessment scores were performed using Student’s *t*-tests and assumed unequal variance. Effect sizes were computed as the standardized mean difference using Hedges’ *g* so as to account for bias caused by small sample size [66].

### Experimental Design

Participants performed a stop signal task during fMRI [17], [67]–[69]. This paradigm has consistently activated regions associated with inhibitory processing, including the middle frontal gyrus and dorsal ACC [46], [68]. The scan session for all participants began at 9 a.m., following at least an 8 hour (overnight) fast. Just prior to the scan, all participants performed an abbreviated version of the task in order to determine their mean reaction time (MRT). Participants were asked to respond as quickly and accurately as possible with a left or right button press when they saw an “X” or an “O” stimulus (i.e., the “go” stimulus), respectively, but to not press either button when they heard a tone (i.e., the “stop” stimulus) that coincided with the presentation of the visual stimuli. The timing of the tone relative to the visual stimulus was manipulated, such that it was either easy or hard for the participant to inhibit a response. Specifically, individualized easy (i.e., tone occurred either 400 or 500 ms prior to MRT) or hard (i.e., tone occurred either at MRT or 100 ms prior to MRT) trials were constructed for each individual. Each trial lasted 1300 ms, or until the participant responded. Trials were separated by a 200 ms interstimulus interval. Participants performed a total of 72 stop trials, which were pseudo-randomized throughout the task, and counterbalanced. A total of six blocks were performed, each containing 48 total trials (12 stop and 36 nonstop trials per block). Task instructions were presented for 12 sec between blocks. All participants received the same number of hard and easy trials; these were unique to each individual, as they were based upon each participant’s prescan MRT.

### MRI

Imaging data were collected with a 3T Signa Excite scanner (GE Medical Systems). FMRI was performed with gradient-recalled echoplanar imaging (TR  =  2000 ms, TE  =  30 ms, flip angle  =  80°, 64×64 matrix, ASSET factor  =  2, 40 2.6-mm ascending interleaved axial slices with a 0.4-mm gap, 256 volumes) [70], [71]. The first four volumes of each run were discarded to allow for T1 saturation. EPI-based field maps were also acquired to correct for susceptibility-induced geometric distortions [72], [73]. A high resolution T1-weighted image (SPGR, TI  =  600 ms, TE  =  min full, flip angle  =  8°, 256×192 matrix, 170 1.2-mm contiguous slices) was obtained for subsequent spatial normalization.

#### Definition of anatomical regions of interest

Regions of interest (ROI) included the anterior cingulate, posterior cingulate (PCC), and middle frontal gyrus (MFG) derived from the Harvard-Oxford Atlas as applied using FMRIB FSL (http://fsl.fmrib.ox.ac.uk/fsl/). The anterior cingulate was further divided into rostral and dorsal subcomponents [74], The rostral ACC, known to project to the limbic striatum [48], was distinguished from the cognitive zone of the dorsal ACC by drawing a 45 degree line from the anterior commissure. The cognitive zone of the dorsal ACC, which projects to executive striatal and prefrontal regions, was defined from this line to a line vertical to the anterior commissure. The MFG ROI was first masked with the MNI template mask, and then eroded by one voxel around its surface in order to avoid potential artifact along the edge of the brain.

### Behavioral analysis

Participants’ inhibition accuracy during the stop signal task, determined as the percentage of trials that were successfully inhibited, was subjected to a repeated measures general linear model with group (CA, AN) as a fixed between-subjects factor, trial difficulty (easy, hard) as a fixed within-subject factor (hard = MRT-0 and MRT-100 trials; easy = MRT-400 and MRT-500 trials) and subjects as random factor. Groups were also compared on prescan MRT and post-error slowing on easy and hard trials.

### MRI statistical analysis

Functional images were preprocessed and analyzed using Analysis of Functional NeuroImages (AFNI) software [75] and R statistical packages (http://www.r-project.org). EPI images were motion-corrected and aligned to high-resolution anatomical images. Time points with isolated head movements not corrected by coregistration were censored from the statistical analysis. Participants with greater than 1 voxel movement were excluded from further analysis. This resulted in the exclusion of one AN participant, leaving a sample of 11 AN participants and 12 comparison adolescents available for group analysis. Statistical analyses were performed using a general linear model (GLM), whereby individual events were modeled using AFNI’s waver function. Task regressors of interest included successfully inhibited trials and failed inhibited trials, both of which were parameterized by difficulty level (i.e., easy, hard), and Go trials, where the participant was expected to make a response. Three motion parameters (rotations) were used as nuisance regressors to account for motion artifact. Given the potential for ventricular widening and sulcal atrophy due to malnutrition in the group with AN, registration to the MNI-152 atlas was performed using FMRIB's Non-linear Image Registration Tool (FNIRT), a part of FSL (http://fsl.fmrib.ox.ac.uk/fsl/). Functional data were scaled to percent signal change (PSC) and smoothed with a 4.2 mm FWHM Gaussian kernel. The PSC map for each individual was visually inspected for outliers before inclusion in group analyses.

For each ROI, a diagnosis (AN, CA) x inhibition accuracy (successful inhibit, failed inhibit) x difficulty (easy, hard) linear mixed effects (LME) analysis in R was performed, with the ROI of interest treated as a search region [76]. Participant was treated as a random effect with diagnosis as the between-group factor, and inhibition accuracy and difficulty were treated as within-subject factors. Age was included as a covariate to control for possible age-related differences in frontal cortex development. The interactions of group x inhibition accuracy and group x difficulty were of primary interest. Small volume family-wise error correction was determined with Monte-Carlo simulations (via AFNI’s 3dClustSim) to guard against false positives, and a cluster threshold of p<0.05 with a peak voxel of p<0.05 was required for significance; the minimum cluster size for each region is provided with the results. Post hoc analyses were conducted using Tukey’s HSD. Exploratory Pearson product-moment correlation coefficients using the mean PSC within each ROI and behavioral measures of interest, log transformed to reduce the influence of outliers, were computed to explore potential correlations. We also performed an exploratory whole brain voxelwise analysis, using the same LME model as performed with the ROIs. To guard against false positives, Monte-Carlo simulations using 3dClustSim indicated that clusters larger than 235 voxels (1880 μL) at a threshold of p<0.05 (with a peak voxel of p<0.05) were considered significant.

## Results

### Demographics and clinical assessments

AN and CA individuals were of similar age and intelligence (Table 1), but as expected, participants with AN had lower BMI and elevated measures of core ED symptoms compared with the CA group. AN participants had a greater score on the BDI relative to CA (*t*(9.1) = 6.2, *p*<0.001, *g* = 2.5), and a significantly greater number of AN participants met criteria for depression relative to CA (*X* ^2^(1, N = 23) = 12.9, *p* = 0.003). There were some performance differences on the WCST: participants with AN (mean ± SD: 12.7±6.4) committed more perseverative errors than CA (7.0±2.1), indicating that the group with AN was less adaptive to cognitive shifts than the CA group (*t*(9.7) = 2.5, *p* = 0.03, *g* = 1.0). There was no difference between groups in the number of categories completed.

**Table 1:** Clinical and demographic characteristics.

Characteristic	AN (N = 11)	CA (N = 12)	T value	DF	P value	Hedge's g
Age (years)	16.0 (2.0) [14.0–19.0]	14.9 (1.8) [12.0–17.0]	1.4	20.2	0.2	0.5
Illness Duration (months)	32.9 (24.1) [10.0–86.0]					
Body Mass Index	16.9 (1.5) [13.1–19.0]	20.8 (1.6) [18.5–23.3]	–6.0	20.9	<0.001	–2.4
% Ideal Body Weight	84.0 (4.6) [77.7–91.9]	104.4 (6.1) [94.8–113.0]	–9.0	20.3	<0.001	–3.6
Age at menarche (years)^a^	12.9 (1.6) [11.0–15.0]	11.7 (0.7) [11.0–13.0]	1.9	9.3	0.08	0.8
BDI^b^	19.4 (9.7) [6.0–39.0]	0.3 (0.9) [0.0–3.0]	6.2	9.1	<0.001	2.5
Current depression (% of total)	39.1%	0%	*X* ^2^ = 12.9	1.0	0.003	
Drive for Thinness (EDI-2)^b^	12.1 (6.2) [1.0–19.0]	0.1 (0.3) [0.0–1.0]	6.1	9.0	<0.001	2.5
Body Dissatisfaction (EDI-2)^b^	10.1 (8.6) [0.0–20.0]	0.0 (0.0) [0.0–0.0]	3.7	9.0	<0.001	1.5
Perfectionism (EDI-2)^b^	9.9 (6.6) [1.0–18.0]	3.2 (2.5) [0.0–8.0]	3.1	11.1	0.01	1.2
Harm Avoidance (TCI)^c^	22.8 (5.8) [12.0–30.0]	6.4 (3.3) [2.0–11.1]	7.6	11.9	<0.001	3.0
Trait Anxiety (STAI)^b^	54.6 (10.5) [40.0–68.0]	23.3 (3.2) [20.0–29.0]	9.1	10.4	<0.001	3.7
WASI - Similarities (T-score)	56.8 (9.2) [44.0–75.0]	57.9 (9.8) [43.0–76.0]	–0.3	21.0	0.8	–0.1
WASI - Matrix Reasoning (T-score)	55.6 (4.0) [51.0–63.0]	56.6 (3.8) [52.0–63.0]	–0.6	20.6	0.6	–0.2
WCST - Perseverative Error (raw)^d^	12.7 (6.4) [5.0–25.0]	7.0 (2.1) [4.0–11.0]	2.5	9.7	0.03	1.0
WCST - Categories Completed (raw)^e^	5.2 (1.7) [1.0–6.0]	5.9 (0.3) [5.0–6.0]	–1.2	8.5	0.3	–0.5
WRAT4 - Reading (SS)^b^	111.0 (15.4) [93.0–145.0]	115.2 (15.5) [98.0–145.0]	–0.6	19.3	0.5	–0.3

Note: Entries are of the form: mean (SD) [min - max]. Statistical comparisons were by means of Welsh t-tests. AN: adolescent females with anorexia nervosa restricting-type; BDI: Beck Depression Inventory; CA: healthy comparison adolescent females; DF: degrees of freedom; EDI: Eating Disorders Inventory; SS: standard score; STAI: State-Trait Anxiety Inventory; TCI: Temperament and Character Inventory; WASI: Wechsler Abbreviated Scale of Intelligence; WCST: Wisconsin Card Sorting Test; WRAT4: Wide Range Achievement Test Revision 4. ^a^Two AN were pre-menarche and were excluded from this measure and one AN and three CA were missing responses for this measure; ^b^one AN was missing responses for this measure; ^c^two AN were missing responses on this measure; ^d^two AN and two CA were missing responses on this measure, and one CA was excluded due to extreme scores; ^e^two AN and two CA were missing responses on this measure.

### Behavioral analysis

**Figure 1:**
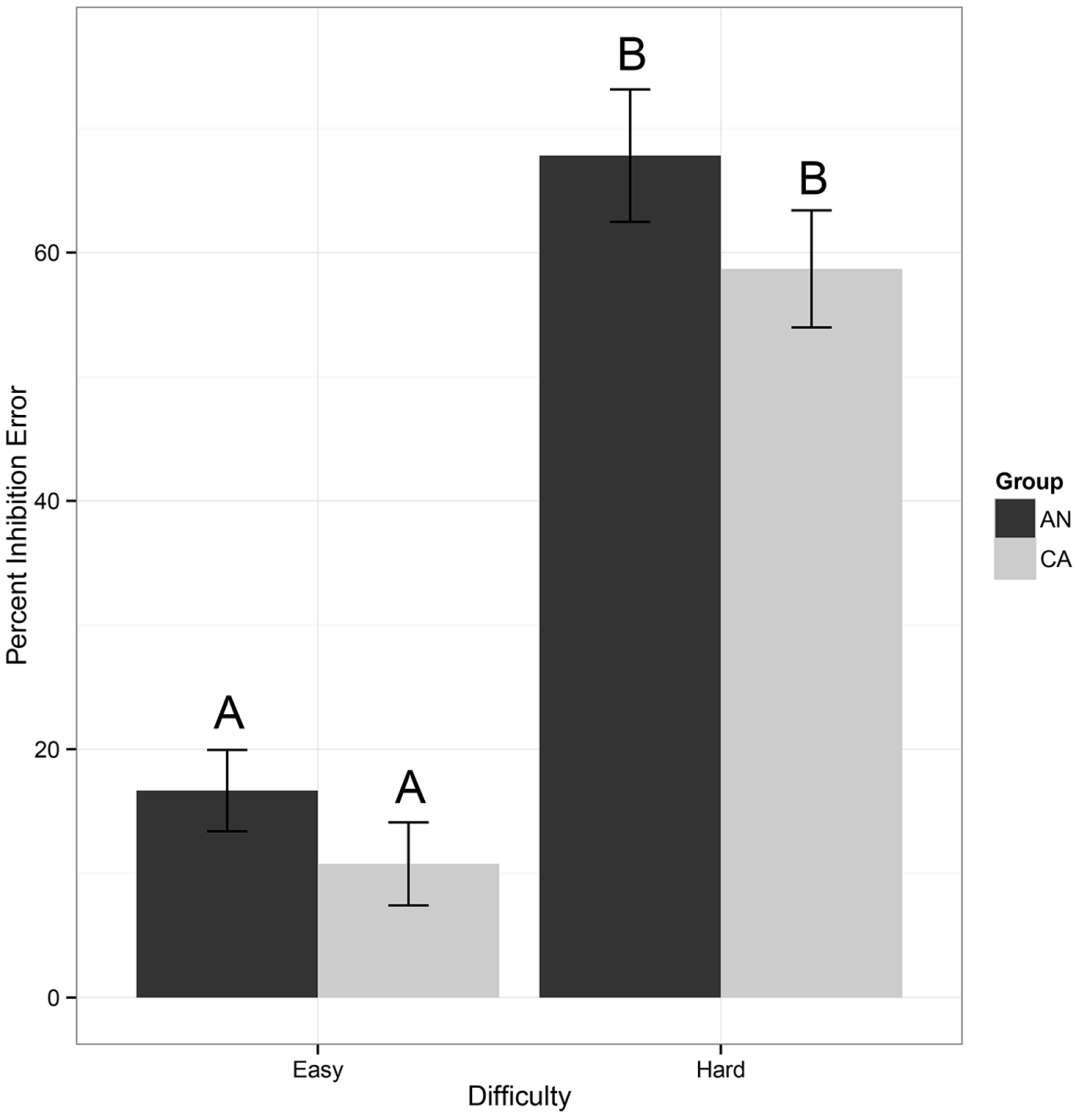
Behavioral accuracy performance on the stop signal task. The percent of inhibition errors (failed inhibits) for easy stop trials vs. hard stop trails for each group. No group or group x trial type differences were revealed for accuracy of performance. Bars with different letters (A vs. B) are significantly different from one another: participants made significantly more errors during the hard stop trials than during the easy stop trials [F(1,21)  =  264,6, p<0.001, g = 6.5]. Error bars represent the standard error for each group. AN: ill adolescents with anorexia nervosa, restricting-type; CA: healthy comparison adolescents.

**Figure 2:**
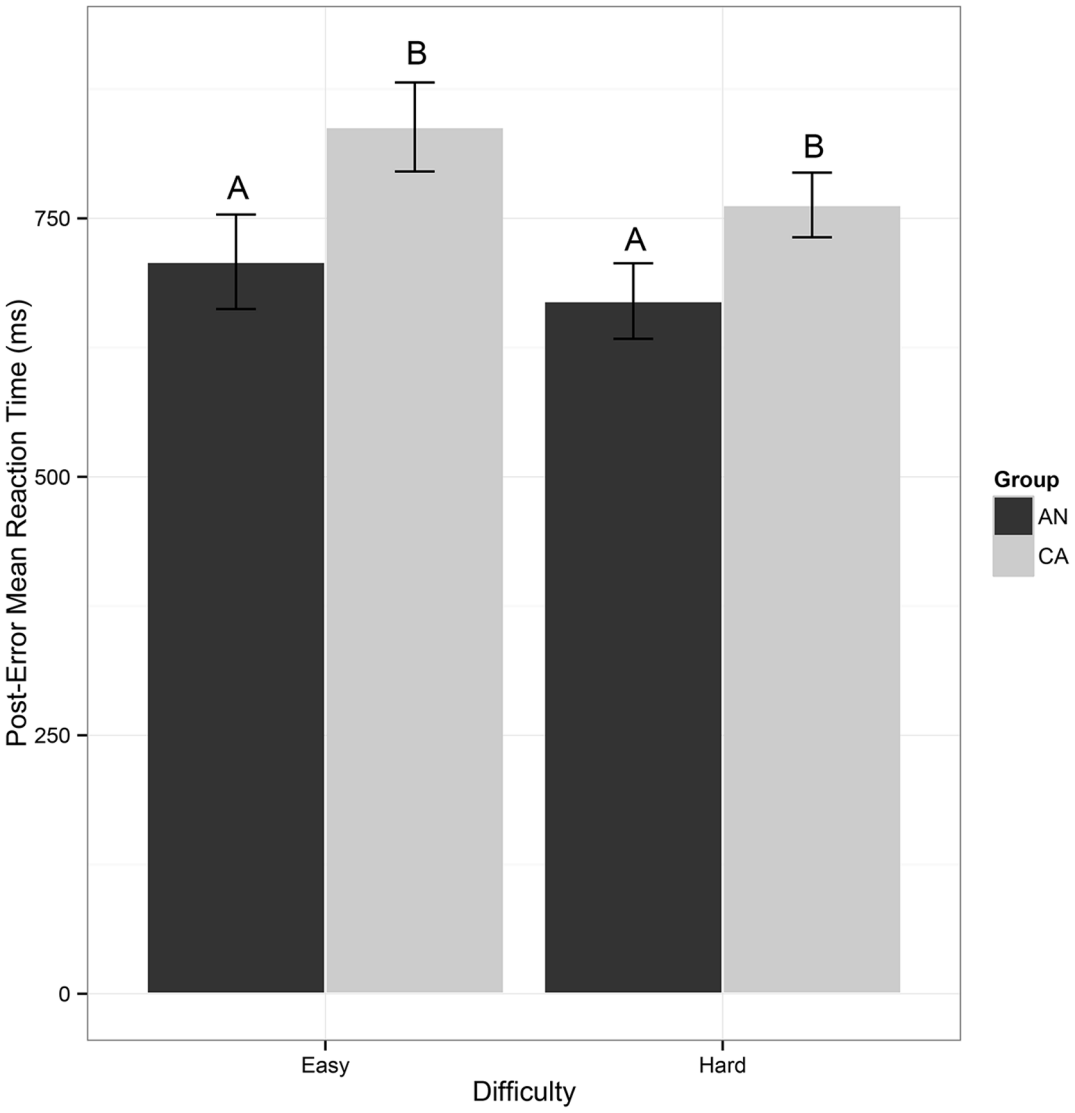
Mean reaction time (MRT) in milliseconds for post-error slowing (trials following a failed inhibition trial) for errors that occurred on easy stop trials and hard stop trials. As represented by the uppercase letters on the barplot, the MRT for post-error slowing was significantly faster [F(1,21)  = 4.6, p = 0.04, g = 0.9] for AN (689.0±135.3 ms) than CA (800.7±133.1 ms). Error bars represent the standard error for each group. AN: ill adolescents with anorexia nervosa, restricting-type; CA: healthy comparison adolescents.

When averaged across all trials, there was no significant difference in the prescan MRT (t(20.89) =  –1.41, p = 0.2, g = –0.6) between AN (608±159 ms) and CA (702 ±162 ms). Overall, participants failed to correctly inhibit behavioral response on 34.4% of total stop trials. Both groups committed more inhibition errors during the hard stop trials (AN: 67.8±17.7% error; CA: mean = 58.6±16.3% error) relative to the easy stop trials (AN: 16.7±10.9% error; CA: 10.8±11.6% error), F(1,21) = 264.6, p<0.001, g = 6.5 (Figure 1). No significant group (F(1,21) = 2.1, p = 0.2, g = 0.6) or group by difficulty (F(1,21) = 0.3, p = 0.6, g = 0.2) effects for inhibition errors were detected, indicating that the two groups were not significantly different in inhibition accuracy during any of the trials. A group x difficulty analysis of reaction time following errors on easy and hard trials demonstrated a main effect of group (F(1,21) = 4.6, p = 0.04, g = 0.9), whereby AN (689.0±135.3 ms) exhibited faster post-error MRTs relative to CA (800.7±133.1 ms). There was also a main effect of difficulty (F(1,21) = 8.9, p = 0.007, g = 1.2); easy stop trials (776.0±161.2 ms) exhibited a slower post-error MRT relative to hard stop trials (718.5±121.6 ms). The group x difficulty interaction was not significant (Figure 2).

### FMRI Analysis: Inhibition-related processing

#### ROI Results

**Figure 3:**
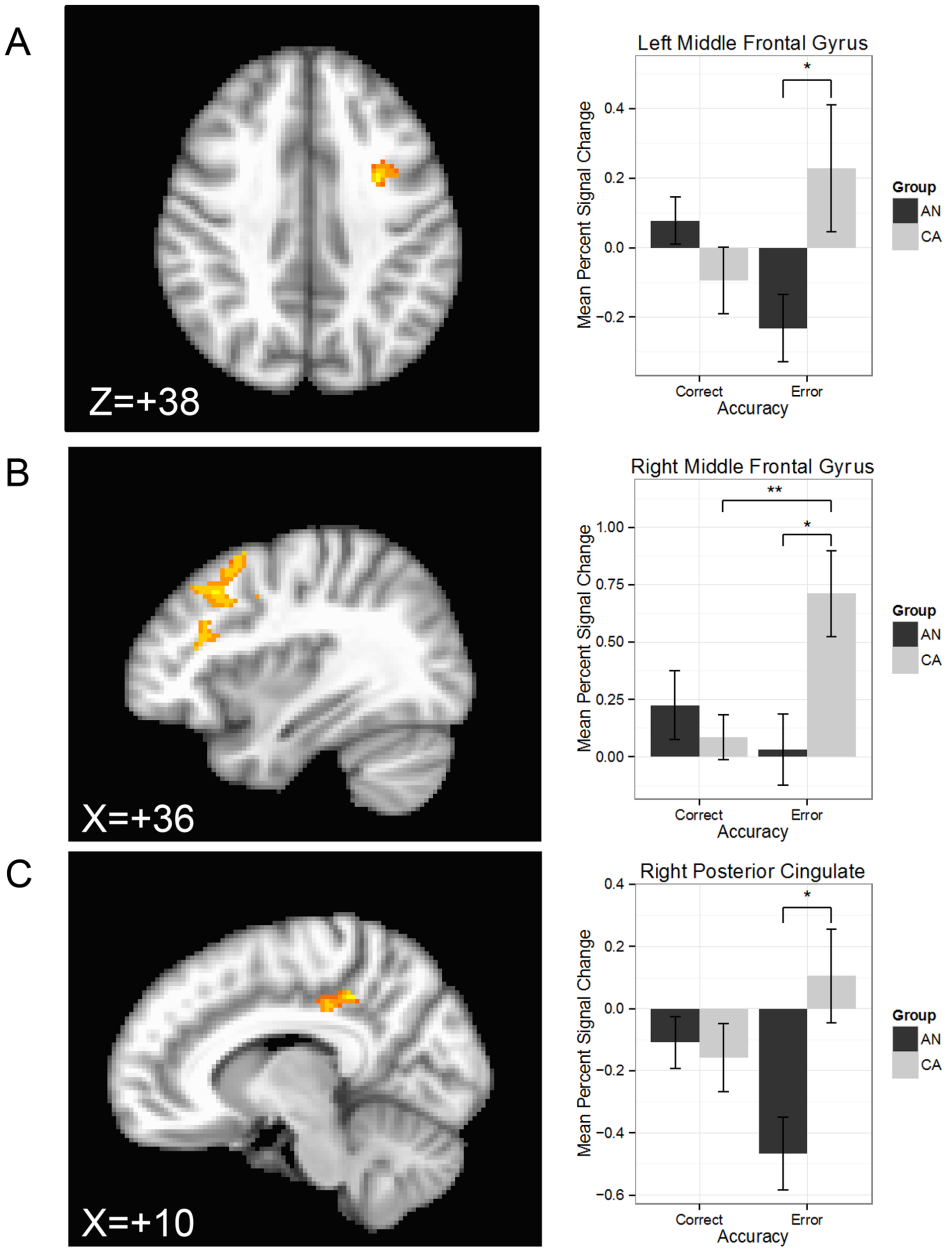
Statistical parametric maps illustrating the location of the interactions for the group x difficulty (easy, hard trials) interaction within regions of interest (left column) and the mean percent signal change within the cluster differentiated by group and condition (right column) for the A) right dorsal anterior cingulate, B) left middle frontal gyrus, C) right middle frontal gyrus, D) left posterior cingulate, and E) left rostral anterior cingulate. Hot colors indicate voxels reflecting a greater response to the group x difficulty interaction. AN: ill adolescent females with anorexia nervosa; CA: control adolescent females; voxel-wise p<0.05; cluster threshold > 392 μL; ***p<0.005.

Regions demonstrating a group x difficulty (easy, hard) interaction included the right dorsal ACC, the bilateral MFG, the left PCC, and the left rostral ACC (Table 2). Post hoc t-tests revealed that within the right dorsal ACC, right MFG, and left PCC, these interactions were driven by a decreased response in AN relative to CA for hard trials (Table 2, Figure 3). Within-group comparisons revealed greater response to easy vs. hard trials in the left MFG and left PCC for AN, and greater response to hard vs. easy trials in the left rostral ACC for CA.

**Table 2:** Analysis of variance results within regions of interest demonstrating an interaction of group (CA, AN) by difficulty (easy, hard).

Analysis of variance									Post hoc comparisons		
					Peak MNI Coordinates						
Region	L/R	BA	Volume (μL)	Min cluster size (μL)	x	y	z	*F*	Contrast	z	p
Dorsal anterior cingulate	R	24	440	392	6	–14	46	9.5	Hard: CA>AN	3.5	0.003
Middle frontal gyrus	L	8/9	2112	680	–28	12	32	13.1	AN: Easy>Hard	2.7	0.04
		9/10	1408		–34	36	28	9.3			
	R	8	1104	688	26	16	38	9.0	Hard: CA>AN	2.8	0.03
Posterior cingulate	L	31	1024	432	0	–38	34	8.7	Hard: CA>AN	3.2	0.007
									AN: Easy>Hard	3.0	0.02
Rostral anterior cingulate	L	24	392	336	–2	28	12	8.0	CA: Hard>Easy	2.9	0.02

Note: BA: Brodmann Area; CA: healthy comparison adolescents; L: left; R: right; AN: adolescents ill with anorexia nervosa, restricting-type.

#### Voxelwise Results

Several clusters demonstrated a group x difficulty interaction, including the postcentral gyrus extending into the PCC, the MFG bilaterally, and several clusters within the occipital cortex (Table 3). Post hoc t-tests revealed additional regions with a similar pattern to the ROI results whereby AN individuals had decreased response for hard trials. Specifically, there was a decreased response in AN relative to CA for hard trials in the posterior insula. Within-group comparisons revealed greater response to easy vs. hard trials in the posterior cingulate, lingual gyrus, orbital frontral cortex and putamen for AN, and greater response to hard vs. easy trials in the posterior insula for CA.

**Table 3:** Voxelwise analysis of variance reporting significant clusters for an interaction of group x difficulty.

			Analysis of variance					Post hoc Comparisons		
Region	L/R	BA	Volume (μL)	x	y	z	F	Contrast	z	p
Postcentral Gyrus/Precentral Gyrus/Posterior Cingulate	B	3/4	18344	12	–40	66	15.8	Easy: AN > CA	3.2	0.01
								AN: Easy > Hard	2.7	0.03
								CA: Hard > Easy	2.6	0.05
Supramarginal Gyrus/Superior Parietal Lobule/Precuneus Cortex	R	7/31/18	14960	22	–66	24	21.6	CA: Hard > Easy	3.0	0.01
								Hard: CA > AN	2.5	0.06
Central Opercular Cortex/Posterior Insula/Planum Temporale	R	14/40/41	10432	50	–8	8	15.7	AN: Easy > Hard	2.4	0.07
								CA: Hard > Easy	2.6	0.05
								Hard: CA > AN	2.7	0.03
Middle Frontal Gyrus/Frontal Pole	L	9/8/6	9112	–28	12	32	13.1	Easy: AN > CA	2.6	0.05
								AN: Easy > Hard	2.8	0.03
Lingual Gyrus/Intracalcarine Cortex	R	18/19/36	6832	24	–52	–2	11.1	CA: Hard > Easy	2.8	0.03
Lingual Gyrus/Temporal Occipital Fusiform Cortex	L	19/37	4632	–24	–62	–10	11.6	AN: Easy > Hard	2.7	0.04
Inferior Lateral Occipital Cortex/Occipital Fusiform Gyrus	R	19/37	3904	44	–70	–14	8.8	Easy: AN > CA	2.6	0.05
								AN: Easy > Hard	4.1	<0.001
Central Opercular Cortex/Planum Temporale/ Posterior Insula	L	40/41/14	3800	–56	–10	6	19	CA: Hard > Easy	2.7	0.03
								Hard: CA > AN	2.4	0.07
Postcentral Gyrus/Precentral Gyrus	L	3/4	3000	–52	–24	46	12.9	CA: Hard > Easy	2.4	0.07
Middle Frontal Gyrus/Superior Frontal Gyrus	R	8/6	2416	26	14	38	9.2	Hard: CA > AN	2.4	0.08
Angular Gyrus	R	39	1952	46	–48	16	12.2	CA: Hard > Easy	2.3	0.10
Frontal Orbital Cortex/Putamen	L	12/25	1944	–16	8	–14	11.9	Easy: AN > CA	2.4	0.08
								AN: Easy > Hard	3.4	0.004
Superior Lateral Occipital Cortex	L	7	1928	–32	–62	36	7.7	n.s.		
Frontal Pole/Paracingulate	L	10/32	1912	–20	60	6	9.8	Easy: AN > CA	2.3	0.09
								AN: Easy > Hard	2.4	0.07
								CA: Hard > Easy	2.3	0.08

Note: BA: Brodmann Area; B: bilateral; L: left; R: right.

### FMRI Analysis: Error-related processing

#### ROI Results

**Figure 4:**
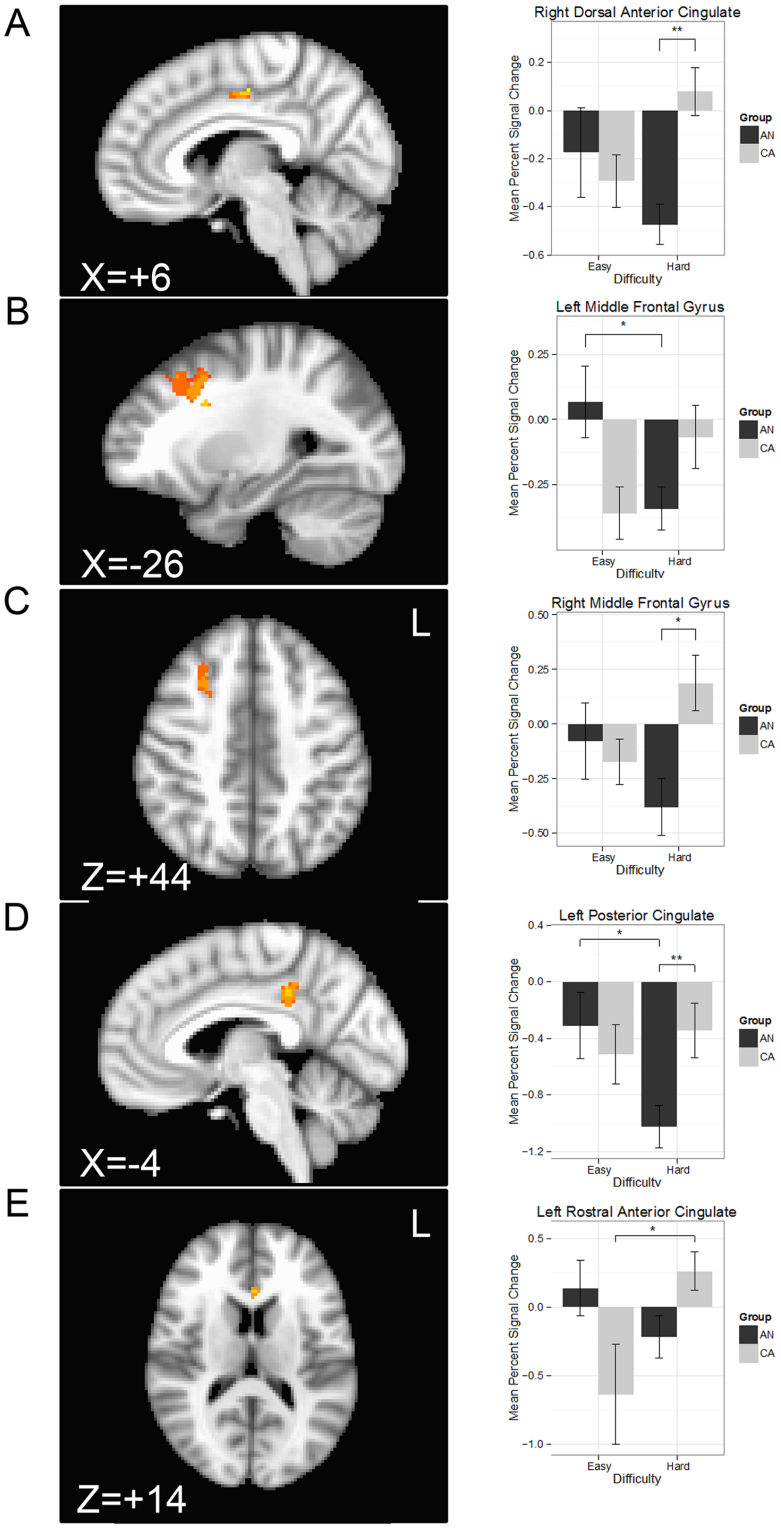
Statistical parametric maps illustrating the location of the interactions for the group x inhibition accuracy (successful inhibit, failed inhibit) interaction within regions of interest (left column) and the mean percent signal change within the cluster differentiated by group and condition (right column) for the A) left middle frontal gyrus, B) right middle frontal gyrus, and C) right posterior cingulate. Hot colors indicate voxels reflecting a greater response to the group x inhibition accuracy interaction. AN: ill adolescent females with anorexia nervosa; CA: control adolescent females; voxel-wise p<0.05; cluster threshold > 688 μL; *p<0.05; ***p<0.005.

A significant group x inhibition accuracy (successful inhibit, failed inhibit) interaction was found within the bilateral MFG and the right PCC for stop trials (Table 4). Post hoc t-tests revealed that the AN showed a reduced response to failed inhibit trials compared to the CA in the bilateral MFG and right PCC (Figure 4). The CA also demonstrated an overall greater response to failed inhibit relative to successful inhibit trials within the right MFG.

**Table 4:** Analysis of variance results within regions of interest demonstrating an interaction of group by inhibition accuracy (successful inhibit, failed inhibit).

Analysis of variance									Post hoc comparisons		
					Peak MNI Coordinates						
Region	L/R	BA	Volume (μL)	Min cluster size (μL)	x	y	z	*F*	Contrast	z	p
Middle frontal gyrus	L	8	744	680	–30	4	40	10.2	Failed inhibition: CA>AN	2.6	0.05
	R	8/9	3688	688	28	4	38	8.1	Failed inhibition: CA>AN	2.7	0.03
									CA: Failed inhibition >Successful inhibition	3.4	0.004
Posterior cingulate	R	31	760	408	10	–40	42	10.1	Failed inhibition: CA>AN	3.8	<0.001

Note: Failed inhibition are trials in which participants failed to inhibit motor response when an auditory stop cue was presented, whereas successful inhibition are trials in which the participant correctly inhibited a motor response when the auditory stop cue was presented. BA: Brodmann Area; CA: healthy comparison adolescents; L: left; R: right; AN: adolescents ill with anorexia nervosa, restricting-type.

#### Voxelwise Results

Significant group x inhibition accuracy effects were found in several clusters (Table 5). These regions included clusters within the occipital gyrus, the supramarginal gyrus, the anterior insula, and the superior frontal gyrus. Consistent with the ROI results for error-related processing, post hoc t-tests revealed that for all regions except the occipital cortex, AN showed a decreased brain response to failed inhibit trials compared to the CA. The CA also demonstrated greater response to failed inhibit relative to successful inhibit trials within these regions and greater activity for correct compared to failed inhibit trials in the occipital regions.

**Table 5:** Voxelwise analysis of variance reporting significant clusters for an interaction of group x inhibition accuracy.

			Analysis of variance					Post hoc Comparisons		
Region	L/R	BA	Volume (μL)	x	y	z	F	Contrast	z	p
Occipital Fusiform Gyrus/Lingual Gyrus/Intracalcarine Cortex	B	19/18/17	16384	–24	76	–4	15.9	CA: Correct > Error	3.5	0.002
Supramarginal Gyrus/Angular Gyrus	R	39/40	3728	–42	36	40	11.2	n.s.		
Anterior Insula	L	13	2968	28	–10	10	10.8	CA: Error > Correct	3.0	0.01
								Error: CA > AN	3.3	0.006
Brain Stem			2504	–10	20	–20	11.7	CA: Error > Correct	3.9	<0.001
								Error: CA > AN	3.8	<0.001
Middle Temporal Gyrus/Parietal Operculum Cortex	L	42/40	2168	42	52	10	12.5	CA: Error > Correct	2.6	0.05
								Error: CA > AN	3.3	0.006
Superior Frontal Gyrus	R	6	2016	–22	–18	60	10.1	CA: Error > Correct	3.4	0.004
								Error: CA > AN	2.8	0.03

Note: BA: Brodmann Area; B: bilateral; L: left; R: right.

#### Exploration of the relationship between ROI BOLD response and WCST

Correlations between BOLD response to the SST and WCST perseverative errors did not survive correction for multiple comparisons. Uncorrected p-value results are presented in Appendix S1 and Figure S1.

## Discussion

Our study yielded two main preliminary results. Consistent with our first hypothesis, currently ill AN adolescents relative to matched adolescents with no history of AN showed less inhibition-related activation within the right MFG, right dorsal ACC, and left PCC as inhibitory demand was increased during a validated stop inhibition task. This finding is consistent with our prior study in adult women recovered from AN [17] that revealed a group (control, RAN) x condition (hard, easy) interaction in the prefrontal cortex, including the MFG, using a whole brain analysis approach. Replication of these findings in ill adolescent AN suggests that altered inhibition-related activity may be related to core behaviors of AN and is not age or disease-state specific. Second, we demonstrated that, compared to CA, AN exhibited less error-related activation in the bilateral MFG and right PCC. Other studies have suggested that inhibitory control in AN may be influenced by error processing. Adults ill with AN have demonstrated reduced dorsal ACC response to commission errors on a flanker task [56] and decreased activation in the ventral anterior cingulate-striato-thalamic loop relative to controls during response shifting, suggestive of altered performance monitoring [19]. Overall, these results suggest alterations in inhibition and error monitoring that may partially explain the ability to inhibit consummatory behavior.

We previously interpreted decreased BOLD response during inhibitory processing to suggest that AN individuals require less inhibitory resources (i.e., neural activation) to maintain behavioral performance as inhibitory load is increased. More experience with cognitive tasks, corresponding to greater task efficiency, can reduce activation [77], whereas inefficient performance can lead to increased activation in clinical populations [78]. In addition to replicating findings of altered MFG brain response in AN, ill adolescent AN revealed decreased BOLD response to hard trials in the right dorsal ACC and left PCC that may reflect impaired representation of task difficulty consistent with the cognitive inflexibility and set-shifting impairment common in AN [23], [30]. For instance, the dorsal ACC has been generally implicated in motor control and response selection, particularly when presented options conflict on several dimensions [50]–[52], [79]–[81], and more specifically it is implicated in response inhibition [53]. Furthermore, although our prior study focused on adults recovered from AN so as to avoid the potentially confounding effects of starvation, it is important to note that altered cognitive control persists after recovery [12]. Thus, decreased dorsal ACC response further supports the hypothesis of more efficient inhibitory control in AN.

To further test the hypothesis that AN individuals have altered inhibitory processing, we extended our prior findings [17] by demonstrating alterations in error-related brain activation, particularly the PCC and bilateral MFG. The PCC, part of the error-processing network, has been implicated in error monitoring for its role in evaluative functions such as monitoring behavior and is specifically thought to be involved in processing feedback to errors [82], [83]. Although we did not find group differences in the ACC as in previous studies of error performance [19], decreased activation in the MFG and PCC in AN adolescents during failed inhibit trials again suggests AN adolescents elicit fewer cognitive resources during error processing, possibly due to either more efficient error detection and correction or to decreased monitoring of errors. Behaviorally, despite performing the stop task with equivalent inhibition accuracy, AN adolescents had reduced post-error slowing (e.g., they responded faster than CA on subsequent trials following an error). Post-error slowing, or the tendency to slow down on trials subsequent to errors, is typical in healthy adults [84], and has been interpreted as evidence that humans monitor their behavior and can detect and compensate for errors. A lack of post-error slowing in AN despite similar baseline reaction times between groups further suggests reduced effort to monitor errors in AN. More studies are needed to better examine the neural response associated with increasing error monitoring complexity.

Overall these results are consistent with previous studies examining cognitive inhibition in adolescent eating disorders and add to a growing literature indicating altered fronto-striatal circuitry underlying inhibitory control in eating disorders. For example, Marsh et al [85] reported that adolescents with bulimia nervosa (BN) have altered self-regulatory control necessary to resolve conflict, characterized by a failure to activate the right inferolateral and dorsolateral prefrontal cortices, posterior cingulate, and putamen during correct responses in conflict trials, suggesting a release of cognitive control that may contribute to disinhibited binge/purge behavior in BN. Similarly, on a Go/No-Go motor inhibition task, a binge eating/purging adolescent group showed significantly greater activation than the healthy comparison group in the bilateral precentral gyri, anterior cingulate cortex, and middle and superior temporal gyri as well as greater activation relative to both comparison and restricting type AN participants in the right dorsolateral prefrontal cortex, suggesting greater effort was required to inhibit behavior in binge/purge subtypes [20]. Taken together, these studies suggest that eating disorder subtypes may be distinguishable in terms of neural correlates of inhibitory control, and that AN and BN may lie on opposite ends of a spectrum of inhibition/disinhibition.

Despite replicating previous findings, the current study is limited by its modest sample size and results are viewed as preliminary, though the limitations of a small sample size are somewhat counteracted by the use of robust statistics and a well-validated cognitive task. One AN participant was taking olanzapine, but re-running the analysis without this subject did not appreciably change the results. We studied patients in the ill state, so it is possible that the effects of malnutrition influenced the results. However, all participants were enrolled in treatment that required adherence to a meal plan for weight-restoration. Given that current findings are consistent with our previous findings in recovered AN, this supports trait-based effects rather than effects of state alone. Adolescents are still undergoing development of limbic and cognitive systems, particularly within frontal regions associated with this task [86]. However, we found similar results to our adult study, supporting the clinical observation of elevated inhibitory control in AN. Versions of the stop signal task have been used in several other related clinical populations, including adolescent depression [87] and OCD [88]. In depressed adolescents, Yang et al [87] reported a decreased response in the bilateral medial frontal gyrus (BA 10) during stop trials, and adolescents with OCD showed decreased response within the DLPFC and dorsal ACC during failed inhibit trials [88]. Given that adolescents with AN often suffer from co-morbid depression and anxiety, it is possible our findings in AN are related to these symptoms. However, we failed to find a relationship between depression or anxiety and BOLD response in our regions of interest (Pearson product-moment correlations all p > 0.05). Lastly, the version of the stop signal task used in this study equated the number of easy and hard trials for each participant to allow for statistical comparison of the BOLD response between trial types of increasing inhibitory demands. Although this allowed for careful examination of the neural circuitry underlying inhibition and error processing, the rate of inhibition failures is not constant, thus limiting our ability to compare current results to existing behavioral studies of the stop signal task.

In summary, these results demonstrate that ill adolescents with AN have altered brain activity during error and inhibitory processing and suggest that clinical symptoms of AN may be driven by altered functioning of brain systems that govern inhibitory control and error processing. These findings also replicate our prior results in recovered AN adults and suggest that altered prefrontal activation during inhibitory processing may represent a behavioral characteristic in AN that is independent of the state of recovery, perhaps reflecting a trait of the disorder. This is consistent with findings that set-shifting impairments in AN persist after recovery [33]. An improved understanding of the neurobiology of this disorder will likely inform development of more effective interventions targeted at modifying the underlying neural substrates where symptoms are encoded.

## Supporting Information

Figure S1 **Correlation of the log transform of WCST perseverative errors with BOLD percent signal change to hard failed inhibit trials in the left middle frontal gyrus.** A) AN (r = -0.8, p = 0.007); B) CA groups (r = 0.09, p = 0.8), z = -2.24, p = 0.03. AN: ill adolescent females with anorexia nervosa; CA: control adolescent females; WCST: Wisconsin Card Sorting Task.
(TIF)

Appendix S1 **Description of the correlation of the log transform of WCST persverative errors with BOLD percent signal change to hard failed inhibit trials in the left middle frontal gyrus at uncorrected p.**
(DOCX)
