# Supplementary figures and images for: Altered BOLD Response during Inhibitory and Error Processing in Adolescents with Anorexia Nervosa
Source: PLoS One. 2014 Mar 20;9(3):e92017. doi: 10.1371/journal.pone.0092017 (PMC3961291; doi:10.1371/journal.pone.0092017)

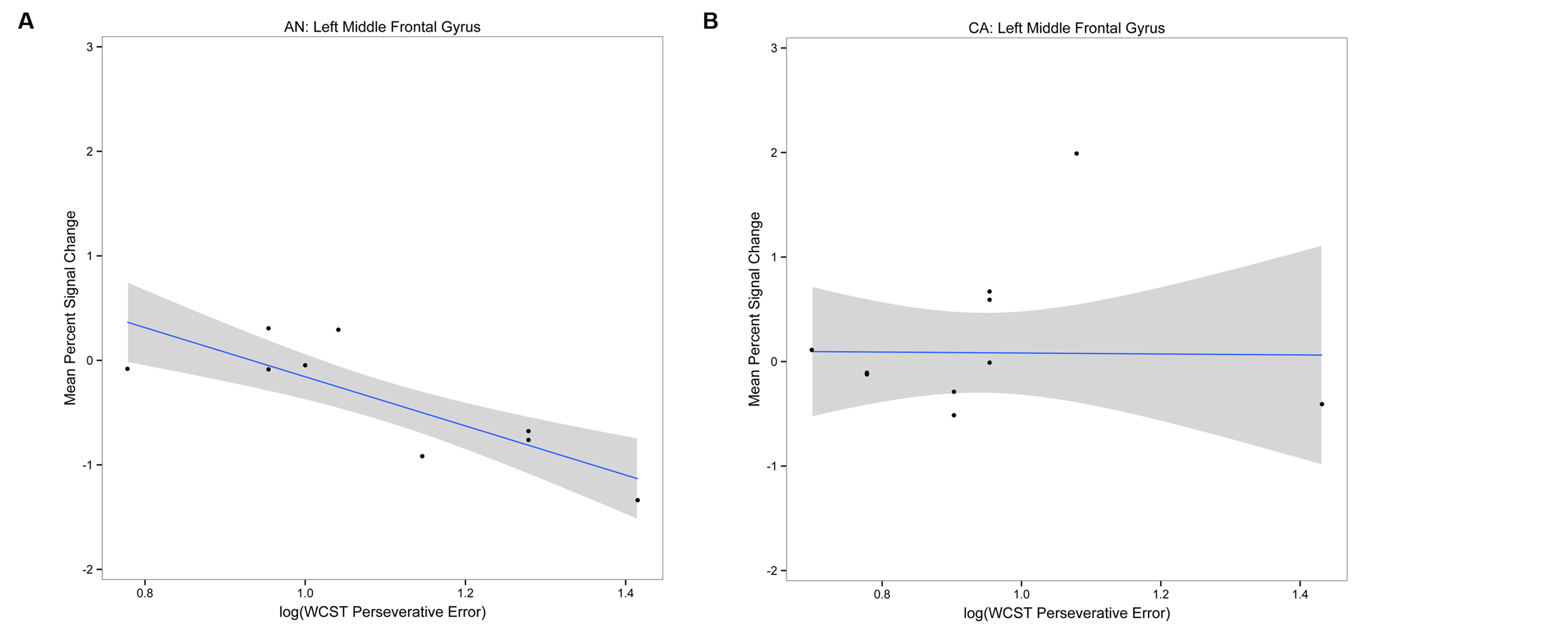

Supplement: Figure S1 — Correlation of the log transform of WCST perseverative errors with BOLD percent signal change to hard failed inhibit trials in the left middle frontal gyrus. A) AN (r = -0.8, p = 0.007); B) CA groups (r = 0.09, p = 0.8), z = -2.24, p = 0.03. AN: ill adolescent females with anorexia nervosa; CA: control adolescent females; WCST: Wisconsin Card Sorting Task. (TIF) [file pone.0092017.s001.tif]
