# Supplementary material for: Altered BOLD Response during Inhibitory and Error Processing in Adolescents with Anorexia Nervosa
Source: PLoS One. 2014 Mar 20;9(3):e92017. doi: 10.1371/journal.pone.0092017 (PMC3961291; doi:10.1371/journal.pone.0092017)
Supplement: Appendix S1 — Description of the correlation of the log transform of WCST persverative errors with BOLD percent signal change to hard failed inhibit trials in the left middle frontal gyrus at uncorrected p. (DOCX) [file pone.0092017.s002.docx]

SI Appendix for

Altered BOLD response during inhibitory and error processing in adolescents with Anorexia Nervosa

Christina Wierenga^a^, Amanda Bischoff-Grethe^a*^, A. James Melrose^a^, Emily Grenesko-Stevens^a^, Laura E. (Zoe) Irvine^a^, Angela Wagner^a^, Alan Simmons^b,a,^, Scott Matthews^b,a^, Wai-Ying Wendy Yau^a^, Christine Fennema-Notestine^a,c^, Walter H. Kaye^a^

^a^Department of Psychiatry, University of California San Diego, La Jolla, CA 92093, USA; ^b^Veterans Affairs San Diego Healthcare System, San Diego, CA 92161, USA; ^c^Department of Radiology, University of California San Diego, La Jolla, CA 92093, USA.

*Corresponding author: Dept of Psychiatry, University of California, San Diego, 9500 Gilman Dr. #0738, La Jolla, CA 92093-0738, USA. Phone: +1-858-246-0604. Fax: +1-858-246-0556. Email: [agrethe@ucsd.edu](mailto:agrethe@ucsd.edu)

### Exploration of the relationship between ROI BOLD response and WCST

There was a significant negative correlation between the BOLD response to hard failed inhibit trials and the log transform of WCST perseverative errors in the left dorsal ACC (r=-0.7, p=0.03), the right dorsal ACC (r=-0.8, p=0.02), and the left MFG (r=-0.8, p=0.007; Figure S1) for AN. AN participants also showed a significant negative correlation to the log transform of WCST perseverative errors and BOLD response to hard correct trials in the left rostral ACC (r=-0.8, p=0.02), the left dorsal ACC (r=-0.8, p=0.02), and the right dorsal ACC (r=-0.8, p=0.02). None of these results survived FDR correction for multiple comparisons. There were no relationships to WCST perseverative errors for any trial type for CA.

The relationship between performance on the WCST and brain activity during error and inhibitory processing for AN adolescents raises the possibility that deficits in set-switching may result from difficulty in overcoming inhibitory control to change behavioral strategies. As described previously, AN individuals demonstrated good stop signal performance, and showed reduced activation to failed inhibit trials compared to CA within the MFG and PCC. This combination suggests that AN may be efficient at error monitoring or devote less attention to errors; however, it is also possible that the reported neural activation pattern is specific to the SST and does not extend to other tasks or behaviors. This raises the question as to whether this brain activation pattern would be seen for more complex error monitoring that may be driven by external events, such as that seen in the WCST, where error processing can reflect self-monitoring of response error or monitoring a change in environmental demands.
